# Hippocampal Satb2 regulates the cognitive function of adult mice through pleiotrophin

**DOI:** 10.1038/s41419-026-08820-z

**Published:** 2026-05-06

**Authors:** Xin-Ren Yu, Yu-Bing Wang, Zhi-Yi Tu, Ying-Ying Wang, Qiu-Xiang Chen, Pin-Xi Xie, Yuan-Yuan Yong, Yan-Yan Wang, Ting-Ting Zhang, Jia-Jun Sun, Tao Chang, Hui-Xiang Yang, Ning-Ning Song, Yu-Qiang Ding, Xuan Zhao, Lei Zhang

**Affiliations:** 1 Department of Anesthesiology, Shanghai Tenth People’s Hospital, Tongji University School of Medicine, Shanghai, 200072 China; 2 Shanghai Yangzhi Rehabilitation Hospital (Shanghai Sunshine Rehabilitation Center), Tongji University School of Medicine, Shanghai, 201619 China; 3 Clinical Center for Brain and Spinal Cord Research, Tongji University, Shanghai, 200092 China; 4 State Key Laboratory of Cardiovascular Diseases and Medical Innovation Center, Shanghai East Hospital, School of Medicine, Tongji University, Shanghai, 200120 China; 5 Affiliated Shanghai Blue Cross Brain Hospital, School of Medicine, Tongji University, Shanghai, 200020 China; 6 Laboratory Animal Center, Fudan University, Shanghai, 200032 China; 7 State Key Laboratory of Brain Function and Disorders, MOE Frontiers Center for Brain Science, Institutes of Brain Science, Fudan University, Shanghai, 200032 China; 8 Huashan Institute of Medicine (HS-IOM), Huashan Hospital, Fudan University, Shanghai, 200040 China

**Keywords:** Developmental disorders, Autism spectrum disorders

## Abstract

The special AT-rich sequence-binding protein 2 (*SATB2*) is associated with human cognitive ability. Mutations in the *SATB2* gene lead to SATB2-associated syndrome (SAS), characterized by severe intellectual disability. SATB2 is mainly expressed in pyramidal neurons in the cerebral cortex and hippocampus, playing a crucial role in cognitive processes. However, the function of SATB2 in the adult hippocampus remains unclear. In this study, we deleted *Satb2* in the CA1 region of the adult mouse hippocampus and observed cognitive impairments along with significant changes in soma and dendrite morphology. Additionally, we identified the growth factor pleiotrophin (PTN) as a downstream target of Satb2, essential for mediating its impact on cognitive functions. Importantly, increasing PTN expression mitigated the morphological and behavioral deficits resulting from *Satb2* deletion in CA1. Our findings highlight the importance of hippocampal Satb2 in regulating cognitive function in adult mice through PTN modulation.

## Introduction

Cognitive dysfunction is a neurological condition characterized by a significant decline in cognitive abilities, including memory, language, reasoning, calculation, attention, orientation, and executive function. It may also manifest with changes in personality, emotion, and behavior [1]. The etiology of cognitive dysfunction is multifaceted, potentially stemming from neurodegenerative diseases, cerebrovascular diseases, brain injuries, infections, metabolic disorders, mental illnesses, drugs, and toxins [2, 3]. Therefore, investigating the pathophysiological mechanisms of cognitive dysfunction is crucial for understanding and treating associated disorders.

SATB2 (special AT-rich sequence-binding protein 2), a transcriptional factor that binds to matrix attachment regions in DNA and recruits chromatin-modifying complexes [4], has been identified as a genetic risk locus for schizophrenia and human cognitive ability through genome-wide association studies [5, 6]. Recent studies also show that genes regulated by SATB2 or encoding SATB2-interacting proteins contribute to human cognitive function [7, 8]. Rare mutations within the *SATB2* locus lead to SATB2-associated syndrome (SAS), characterized by severe learning difficulties and intellectual disability [9, 10], highlighting the crucial role of SATB2 in cognitive function. Previous research demonstrated that mice lacking the *Satb2* gene in the entire neocortex and hippocampus from embryonic or postnatal stages exhibit learning and memory deficits [11, 12], while the role of Satb2 in the adult hippocampus remains largely unclear.

Pleiotrophin (PTN) belongs to a recently identified family of heparin-binding growth factors, expressed in various tissues during embryonic development [13]. Its functions encompass supporting brain development and homeostasis, as well as tissue regeneration, contingent upon context-specific cell types and receptor diversity [14]. PTN specifically binds to chondroitin sulfate of proteoglycan receptors, finely modulating crucial biological processes of cell activity, such as proliferation, growth, differentiation, and migration [15, 16]. Furthermore, a study has shown that PTN mitigates age-related decline in adult hippocampal neurogenesis and cognitive impairment [17]. Nonetheless, the interaction between Satb2 and the regulation of PTN expression in cognitive processes remains uncertain.

In this study, we investigated the impact of selectively deleting the *Satb2* gene in the hippocampal CA1 field of adult mice on their behaviors. Our findings reveal that mice lacking *Satb2* in CA1 display cognitive dysfunction and hyperactivity. Mechanistically, we show that Satb2 influences the cognitive performance of adult mice by upregulating PTN.

## Materials and methods

### Animals

Adult male C57BL/6J mice (6–8 weeks old) were procured from Shanghai Bikai Keyi Biotechnology Co., Ltd. (Shanghai, China). *Satb2*^flox/flox^ mice utilized in this investigation were used as previously documented [11, 18]. The animals were housed in a regulated environment with temperature maintained between 20 and 26 °C and a humidity level ranging from 30 to 70%. They were provided with food and water ad libitum and kept under a 12-h light-dark cycle. No randomization was used for the allocation of animals to experimental groups. No blinding was done for group allocation during the experiments or when assessing the outcome.

### Stereotaxic viral injection

*Satb2*^flox/flox^ mice were anesthetized using isoflurane and placed in a stereotaxic apparatus (RWD, Shenzhen, China). Following craniotomy, the mice received bilateral injections of 250–500 nL of viruses into the CA1 region (coordinates relative to bregma: anterior/posterior: −2.0 mm, medial/lateral: ±1.5 mm, dorsal/ventral: −1.5 mm). The injection needle was kept in place for an additional 10 min post-injection to aid virus diffusion before being slowly removed. Subsequently, the mice were allowed to recover in their home cages for 3–4 weeks before undergoing behavioral tests or morphological experiments. The viruses used, namely AAV2/9-hSyn-EGFP-3×flag-pA (titer: 1.19E + 13 V.G./ml), AAV2/9-hSyn-EGFP-P2A-iCre-pA (titer: 1.57E + 13 V.G./ml), and AAV2/9-hSyn-PTN-WPRE-pA (titer: 2.27E + 13 V.G./ml), were purchased from Shanghai Taitool Bioscience Co., Ltd. (Shanghai, China). Undiluted viral solution was administered for behavioral tests, whereas a 1:1000 dilution in phosphate-buffered saline (PBS) was used for neuron morphological analysis.

### Histological analysis

Mice were perfused with 4% paraformaldehyde (PFA; Sigma, Saint Louis, MO, USA) before their brains were dissected and postfixed in 4% PFA overnight at 4 °C. Brain slices, 35-μm thick, were obtained using a cryostat (CM1900, Leica, Deer Park, IL, USA).

For immunofluorescent staining, brain slices were subjected to antigen retrieval in 10 mM sodium citrate (pH 6.0) for 5 min at 95 °C. The slices were then incubated overnight at 4 °C with rabbit anti-Satb2 antibody (1:1 000; ab92446, Abcam, Waltham, MA, USA) and mouse anti-GFP antibody (1:1 000; A11120, Invitrogen, Carlsbad, CA, USA). Subsequently, they were treated with biotinylated horse anti-rabbit IgG (1:500; Jackson ImmunoResearch, West Grove, PA, USA) and Alexa Fluor 488 donkey anti-mouse IgG (1:1 000; Invitrogen) for 3 h at room temperature, followed by incubation with streptavidin-Cy3 (1:1 000; Jackson ImmunoResearch) for 1 h and counterstaining with Hoechst 33258 (1:1 000; Sigma) for 10 min at room temperature. Imaging was done using a confocal microscope (FV3000; Olympus, Tokyo, Japan).

For in situ hybridization (ISH), an RNA probe against PTN was synthesized based on the Allen Brain Atlas (http://www.brain-map.org). ISH was carried out as previously described [19]. RNA probes labeled with digoxigenin-UTP (Roche, Basel, Switzerland) were transcribed in vitro. The mRNA signals on 20-μm-thick brain slices were visualized using nitro-blue tetrazolium chloride and 5-bromo-4-chloro-3-indolyl phosphate (Macklin, Shanghai, China). Each ISH experiment was conducted with three animals per group. Imaging was performed using a Nikon Eclipse 80i microscope (Tokyo, Japan).

### Analysis of neuronal morphology

Brain slices (35-μm thick) were obtained 3 to 4 weeks post intracranial viral injections. Confocal imaging of individual CA1 pyramidal neurons was performed using a 60× oil objective lens on a microscope (FV3000, Olympus) at a resolution of 1024 × 1024 pixels. Soma size was determined by outlining the cell body and calculating the area in square micrometers. Dendrites were traced to measure total dendritic length as described previously [20]. For Sholl analysis, concentric circles with 10 μm differences in radius were drawn around the soma, and the number of intersecting dendrites per circle was quantified using ImageJ software. All morphological analyses were conducted in a double-blinded fashion.

### Western blotting

Hippocampal tissues were dissected and lysed with RIPA buffer (#89901; Invitrogen) to extract proteins, whose concentration was determined using the BCA assay. The protein samples were mixed with 5× loading buffer, separated on a 12.5 to 15% SDS-polyacrylamide gel, and transferred onto nitrocellulose membranes. Following blocking with 5% fat-free milk, the membranes were incubated overnight at 4 °C with primary antibodies, then with HRP-conjugated goat anti-rabbit secondary antibodies (1:1 000; LF102, Epizyme, Shanghai, China) for 1 h at room temperature. Antibodies used included rabbit anti-PTN (1:500; ab79411, Abcam) and rabbit anti-β-actin (1:2 000; #4970, Cell Signaling Technology, Danvers, MA, USA). Protein bands were visualized using enhanced chemiluminescence reagents (#1525703, Millipore, Hong Kong, China).

### Cleavage under targets and release using nuclease (CUT&RUN) analysis

The CUT&RUN assay [21] was conducted using the Hyperactive pG-MNase CUT&RUN Assay Kit (HD101; Vazyme, Nanjing, China) following the manufacturer’s protocol for PCR/qPCR. Initially, concanavalin A-coated magnetic beads (ConA beads) were introduced to resuspend cell nuclei and incubated at room temperature for binding. Subsequently, the nonionic detergent digitonin was utilized for membrane permeabilization. Rabbit anti-Satb2 antibody (ab92446, Abcam) or rabbit IgG (#2729, Cell Signaling Technology) were applied and left to incubate overnight at 4 °C.

Following washing steps, the pG-MNase fusion protein was introduced and allowed to incubate for 90 min at 0 °C to cleave the specified genomic regions. The hyperactive pG-MNase facilitated Ca^2+^-dependent cleavage and release of DNA fragments bound by the Satb2 protein. Subsequently, DNA extraction was carried out using Buffer GDP for qPCR as described previously [22, 23]. The qPCR primers utilized were as follows: PTN_PR-F: CACCTGCAGTGAGCTAGACC; PTN_PR-R: GTCCCTCCCAGGACTCATCT; PTN_IN-F: AACCAGCATGGTTGAGTCCA; PTN_IN-R: AGACTGGGCATGGAACAGTG; PTN_EN-F: TCACGGTGTGGGAGGATTTG; PTN_EN-R: CTTAGGGGTGAGTGCCTGTG.

### Behavioral tests

Behavioral tests were conducted during the light cycle, preceded by a minimum of 30-min acclimatization of mice to the experimental room. A one-week interval was maintained between successive behavioral trials.

#### Open-field test

Mice were assessed for locomotion in a novel environment following established protocols [24]. The open-field apparatus, measuring 40 cm in length, 40 cm in width, and 30 cm in height, was used. Over a 10-min period, mouse activity was video recorded from above the apparatus and subsequently analyzed using Anymaze software (Stoelting, San Diego, CA, USA). Parameters such as total distance moved, average velocity, ambulatory time, entries into the central zone, and time spent in the central area were quantified. The equipment was sanitized with 70% ethanol between each trial, and the testing room was illuminated with a brightness level of approximately 200 lux.

#### Novel object recognition test

In the novel object recognition test, a small arena measuring 30 cm × 30 cm was utilized to facilitate object exploration and expedite mice habituation. The arena was enclosed by a black screen on three sides to restrict spatial cues and prevent spatial biases. During the training phase, two identical Lego blocks were positioned on the right and left sides of the arena. Mice were placed in the arena center, allowed to explore freely for 10 min, and then returned to their home cages. Subsequently, one of the objects was substituted with a novel one differing in color and shape 2 h later, and mice were given another 10-min exploration period. Animal behaviors during the test were recorded and monitored via a top camera, analyzed using SMART software, and manually confirmed. The discrimination index was computed as (time spent exploring the novel object – time spent exploring the familiar object) divided by (time spent exploring the novel object + time spent exploring the familiar object). Exploration behaviors such as sniffing, touching (>1 s), and staring at the objects were considered [25].

#### Three-chamber social interaction test

A three-chamber social interaction assay was conducted in a rectangular plexiglass box (90 cm length × 50 cm width × 30 cm height) divided into three chambers [26, 27]. The subject mouse underwent a 10-min habituation period in the arena with two empty cups in the lateral chambers. Subsequently, a novel object (a ball) and a C57BL/6J WT mouse (Stranger 1, S1) were placed in the cups. The barriers were then removed, allowing the subject mouse to freely explore for 10 min in the social preference test. Time spent on investigation was quantified, and preferences for the ball and S1 were calculated: Ball preference % = (time spent exploring the ball)/(time spent exploring the ball + time spent interacting with S1) × 100; S1 preference % = (time spent interacting with S1)/(time spent exploring the ball + time spent interacting with S1) × 100. The social interaction index was calculated by the following formula: (time spent interacting with S1 – time spent exploring the ball)/(time spent interacting with S1 + time spent exploring the ball). In the social recognition test, a novel WT mouse (Stranger 2, S2) replaced the ball, and the subject mouse explored freely for 10 min. S2 preference was calculated as follows: S2 preference % = (time spent interacting with S2)/(time spent interacting with S1 + time spent interacting with S2) × 100. The social interaction index was calculated by the following formula: (time spent interacting with S2 – time spent interacting with S1)/(time spent interacting with S2 + time spent interacting with S1). Investigation time recordings began when the test mice approached within 2 cm of the cups.

#### Spontaneous alternation test

Testing was conducted using a Y-shaped maze (21 cm × 7 cm × 15.5 cm) with three light-colored, opaque arms positioned at 120° angles from each other. Prior to testing, the Y-maze was cleaned using ethanol spray and paper towels. The experimental procedure consisted of a single 5-min trial where mice were allowed to freely explore all three arms of the Y-maze. Mice attempting to climb the maze walls were promptly and gently returned to the arm they exited from to maintain trial integrity. The start arms were randomly assigned to minimize potential bias. The operator was blinded to the test mouse group. An alternation was defined as the mouse sequentially entering all three arms. The memory index was calculated by counting the sequential entries into the three distinct arms (labeled as a, b, and c) and dividing this number by the total potential alternations (total arm entries – 2) [28].

#### Spatial memory test

Spatial reference memory was assessed in mice by conducting a Y-maze test where one arm was blocked during training, designated as the novel arm. Following a 1-h interval, the mouse was reintroduced to the maze with the blockage removed. The mouse explored the maze freely for 5 min, and entries into each arm along with the time spent in each arm were documented [29].

### Statistical analysis

Each experiment was repeated at least three times. All data were tested for normal distribution and homogeneity of variance, and all data met the assumptions of the tests. Statistical analyses were conducted using GraphPad Prism software, version 10.0. *P* values were determined through a two-way ANOVA with Sidak’s multiple comparisons test, a one-way ANOVA with Dunnett’s multiple comparisons test, or a two-tailed Student’s *t*-test. Data were expressed as mean ± standard error of mean (S.E.M). Statistical significance was denoted as *P < 0.05, **P < 0.01, ***P < 0.001, ****P < 0.0001, while n.s. indicated *P* > 0.05 (non-significant). The sample size and *P* value for each graph was stated in the figure legends. No statistical methods were used for the sample size estimate.

## Results

### Expression of Satb2 is exclusive to CA1 in the hippocampus

**Fig. 1:**
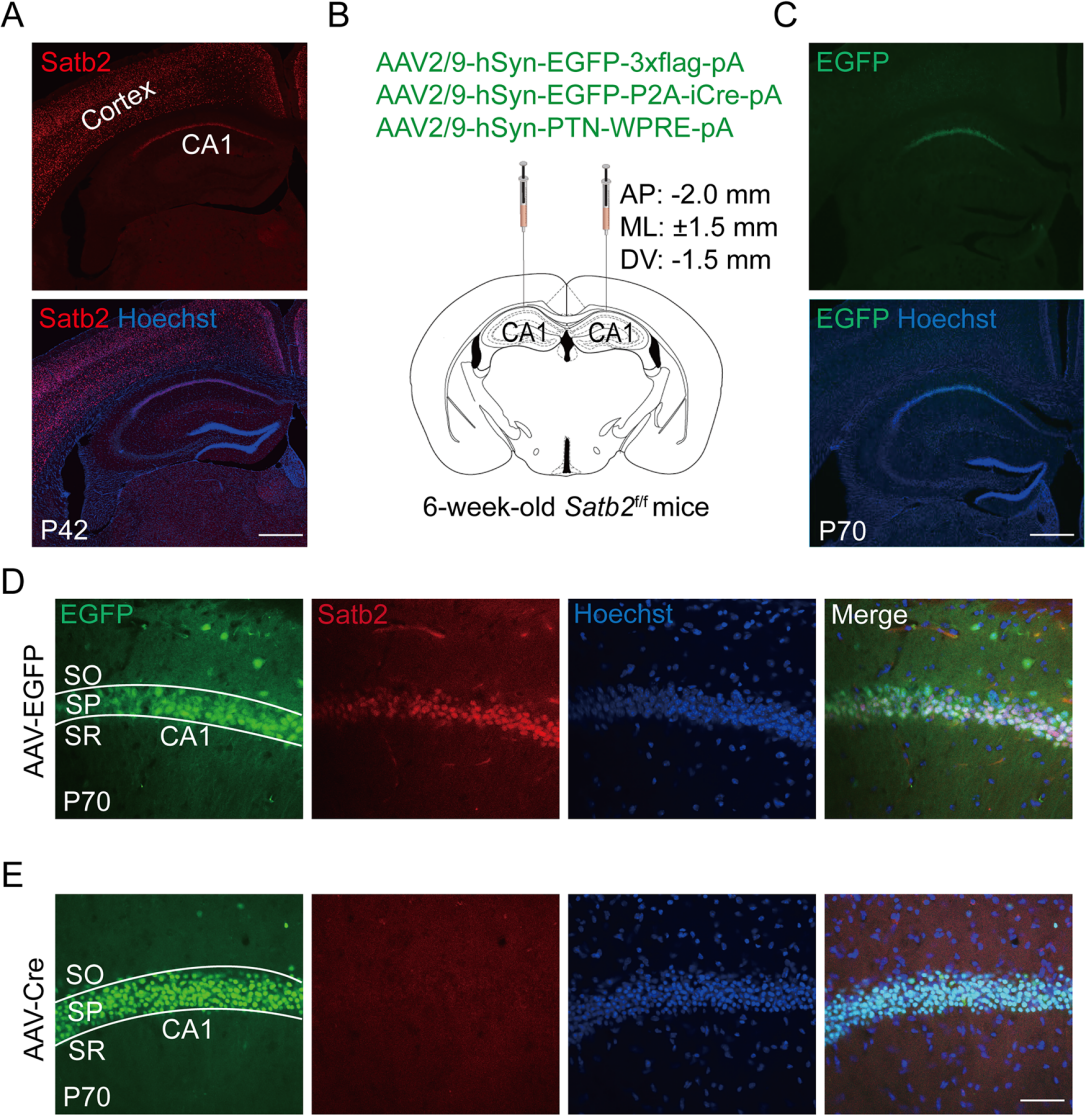
Satb2 is exclusively expressed in the CA1 within hippocampus. **A** Satb2 expression is observed in the cerebral cortex and hippocampal CA1 of P42 wild-type (WT) mice, with cell nuclei stained using Hoechst 33258. Scale bar = 100 μm. **B** Diagram illustrating the viral injection sites in the bilateral hippocampal CA1 of *Satb2*^flox/flox^ mice based on the provided coordinates. **C** EGFP expression following viral infection in the CA1 region of *Satb2*^flox/flox^ mice, with cell nuclei counterstained with Hoechst 33258. Scale bar = 100 μm. **D** Representative images depicting EGFP and Satb2 immunoreactivity in the CA1 of *Satb2*^flox/flox^ mice infected with AAV-EGFP. **E** Representative images showing *Satb2* knockout in the CA1 of *Satb2*^flox/flox^ mice infected with AAV-Cre. CA1 cornu ammonis 1, SO stratum oriens, SP stratum pyramidalis, SR stratum radiatum. Scale bar = 10 μm in (**D**, **E**).

Accumulating evidence indicates a crucial role of Satb2 in the development of the cerebral cortex [18, 30–33]. However, the distribution and function of Satb2 in other brain regions, such as the hippocampus, remain unclear. Immunostaining against Satb2 protein on brain slices from adult mice revealed exclusive expression of Satb2 in the CA1 region of the hippocampus, with no expression in the CA2, CA3, or DG regions, despite abundant expression across all layers of the cerebral cortex (Fig. 1A). To assess the association between Satb2 expression in CA1 and cognitive function in adult mice, adeno-associated viruses (AAVs) carrying enhanced green fluorescent protein (EGFP) alone or EGFP with Cre recombinase were bilaterally injected into the hippocampal CA1 of adult *Satb2*^flox/flox^ mice (Fig. 1B). Our findings demonstrated strong and specific expression of AAV-EGFP in the CA1 region 28 days post-infusion (Fig. 1C). Injection of AAV-EGFP-P2A-iCre (AAV-Cre) confirmed *Satb2* deletion through immunostaining, showing no Satb2 expression in the virus-infected (EGFP-positive) area (Fig. 1E), while Satb2 expression remained largely intact with AAV-EGFP injection (Fig. 1D). These results indicate specific expression of Satb2 in the CA1 region of the hippocampus and highlight viral injection of Cre recombinase into CA1 as a reliable method to target hippocampal *Satb2* in adult mice.

### Loss of Satb2 in the hippocampal CA1 causes cognitive deficits in adult mice

**Fig. 2:**
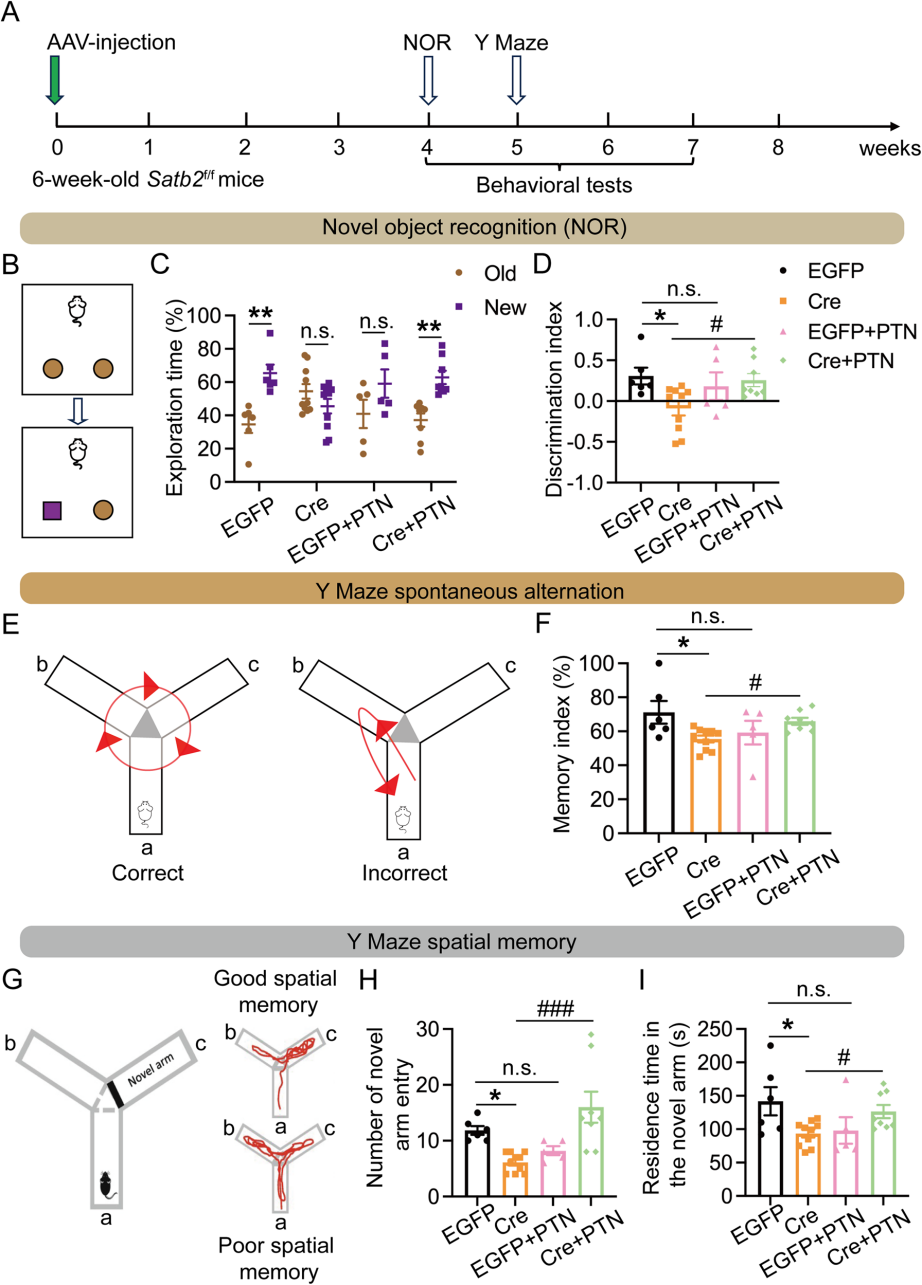
Deletion of *Satb2* in CA1 causes memory deficits which can be ameliorated by overexpression of PTN. **A** A timetable for AAV injection, novel object recognition (NOR) test, and Y-Maze test. **B** A schematic representation of the NOR test. **C** Percentage of time spent exploring in the NOR test. **P < 0.01; n.s. non-significant. **D** Discrimination index in the NOR test. **E** Assessment of spontaneous alternation in the Y-Maze. Left: Correct schematic diagram depicting a high rate of consecutive arm entries. Right: Incorrect schematic diagram showing a higher frequency of repeated entries into the same arm. **F** Memory index in the Y-Maze spontaneous alternation test. **G** Left: Evaluation of spatial memory using the Y-Maze with a blocked arm. Right (top): Mice with good spatial memory will preferentially enter the previously unexplored arm during testing. Right (bottom): Mice with poor spatial memory will exhibit no specific preference. **H** Novel arm entry times in the Y-Maze spatial memory evaluation. **I** Residence time in the novel arm during the Y-Maze spatial memory assessment. Sample sizes: *n* = 6 mice (four males, two females) for AAV-EGFP; *n* = 10 mice (six males, four females) for AAV-Cre; *n* = 5 mice (four males, one female) for AAV-EGFP + PTN; *n* = 8 mice (six males, two females) for AAV-Cre + PTN. A two-way ANOVA with Sidak’s multiple comparisons test or a one-way ANOVA with Dunnett’s multiple comparisons test: EGFP vs Cre, *P < 0.05, **P < 0.01; Cre vs Cre + *P*TN, ^#^*P* < 0.05, ^###^*P* < 0.001; n.s. non-significant. Data were presented as mean ± SEM.

The hippocampus is known for its crucial role in learning and memory processes. Our study aimed to investigate the impact of reduced Satb2 expression on cognitive functions in adult mice. Various behavioral tests were conducted to assess cognitive abilities. Novel object recognition (NOR) test was performed four weeks post viral injection to evaluate novelty recognition (Fig. 2A, B). Results indicated that mice infected with AAV-EGFP showed a significant preference for the novel object (Fig. 2C, D). In contrast, mice infected with AAV-Cre did not display a preference between old and novel objects (Fig. 2C, D), indicating a deficiency in recognition memory.

To investigate potential impacts on spatial working memory, a spontaneous alternation test was conducted using the Y-maze (Fig. 2A, E). Spontaneous alternation, indicative of intact working memory, was assessed by allowing mice to freely explore all three arms of the maze, leveraging rodents’ natural inclination to investigate new areas [34]. Our findings revealed a significant reduction in the memory index (time) in AAV-Cre-infected mice compared to AAV-EGFP-infected mice, irrespective of gender, indicating impaired spatial working memory (Fig. 2F). Furthermore, spatial reference memory, associated with the hippocampus, was evaluated by placing mice in the Y-maze with one arm closed during training (Fig. 2G). Following a 1-h inter-trial interval, mice were expected to remember the unexplored arm and exhibit a preference for it. AAV-Cre-infected mice exhibited notably fewer entries and less time spent in the novel arm compared to AAV-EGFP-infected mice (Fig. 2H, I), suggesting deficits in spatial reference memory.

**Fig. 3:**
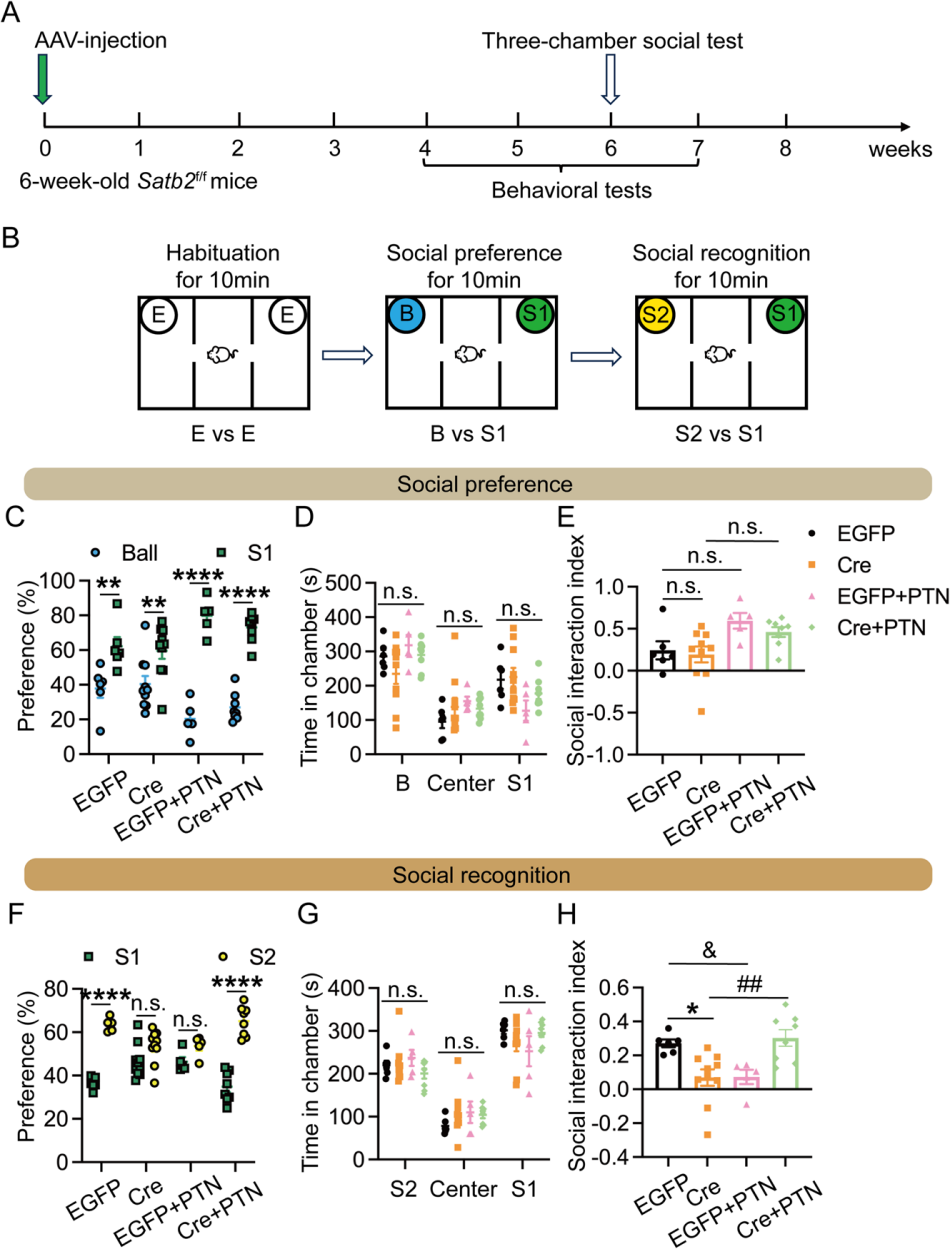
Deletion of *Satb2* in CA1 causes social behavioral deficits which can be ameliorated by overexpression of PTN. **A** Schedule for AAV injection in the CA1 region of postnatal 6-week-old *Satb2*^flox/flox^ mice and subsequent social interaction behavioral test. **B** Experimental design of the three-chamber social behavior test, including habituation, social preference, and social recognition phases. Key: E, empty cup; B, ball; S1, stranger mouse 1; S2, stranger mouse 2. **C** Quantitative analysis of social preference test results across the four experimental groups. **P < 0.01, ****P < 0.0001; n.s. non-significant. **D** Quantification of time spent in each chamber during the social preference phase. n.s. non-significant. **E** Social interaction index comparison among the four experimental groups during the social preference phase. n.s. non-significant. **F** Social recognition test quantification in four groups of mice. ****P < 0.0001; n.s. non-significant. **G** Quantification of time spent in each chamber during the social recognition stage. n.s. non-significant. **H** Social interaction index comparison among the four groups of mice during the social recognition stage. Statistical comparisons: EGFP vs Cre, *P < 0.05; Cre vs Cre + PTN, ^##^*P* < 0.01; EGFP vs EGFP + PTN, ^&^*P* < 0.05; n.s. non-significant; analyzed using two-way ANOVA with Sidak’s multiple comparisons test (**C**, **D**, **F**, **G**) or one-way ANOVA with Dunnett’s multiple comparisons test (**E**, **H**). Sample sizes: *n* = 6 mice (four males, two females) for AAV-EGFP; *n* = 10 mice (six males, four females) for AAV-Cre; *n* = 5 mice (four males, one female) for AAV-EGFP + PTN; *n* = 8 mice (six males, two females) for AAV-Cre+PTN. Data were presented as mean ± SEM.

The impact of Satb2 deletion on hippocampus-dependent social interaction behaviors was further investigated through a three-chamber test conducted six weeks post viral injection into the CA1 (Fig. 3A). The test comprised habituation, social preference, and social recognition stages, each separated by a 1-h interval (Fig. 3B). During habituation, mice were allowed to freely explore two chambers with empty cups for 10 min. In the social preference phase, a cup with a wild-type mouse (stranger 1, S1) was placed in one chamber, while a cup with a ball was placed in the other. Investigation times around the cup with the novel mouse versus the ball were quantified. Results indicated that both AAV-EGFP-infected and AAV-Cre-infected mice showed a preference for exploring the cup with the novel mouse over the ball (Fig. 3C), suggesting a preference for interacting with live mice compared to inanimate objects. Furthermore, both groups spent a similar amount of time in each chamber (Fig. 3D), and their social interaction index was comparable (Fig. 3E). In the social recognition test, a novel mouse (stranger 2, S2) was introduced, and it replaced the ball. The difference in exploration time between the familiar mouse (S1) and the novel mouse (S2) was quantified over 10 min. AAV-EGFP-infected mice predominantly explored the cup with the novel mouse (S2) over the familiar mouse (S1), whereas AAV-Cre-infected mice showed similar exploration times for both S1 and S2 mice (Fig. 3F), indicating impaired social recognition in AAV-Cre-infected *Satb2*^flox/flox^ mice. Also, both AAV-EGFP-infected and AAV-Cre-infected mice spent comparable amounts of time among the three chambers (Fig. 3G). The social interaction index was significantly lower in AAV-Cre-infected mice compared to AAV-EGFP-infected mice (Fig. 3H). These behavioral findings collectively support the involvement of Satb2 in cognitive functions in adult mice, specifically in the hippocampal CA1 region.

### Loss of Satb2 in the hippocampal CA1 causes hyperactivity in adult mice

**Fig. 4:**
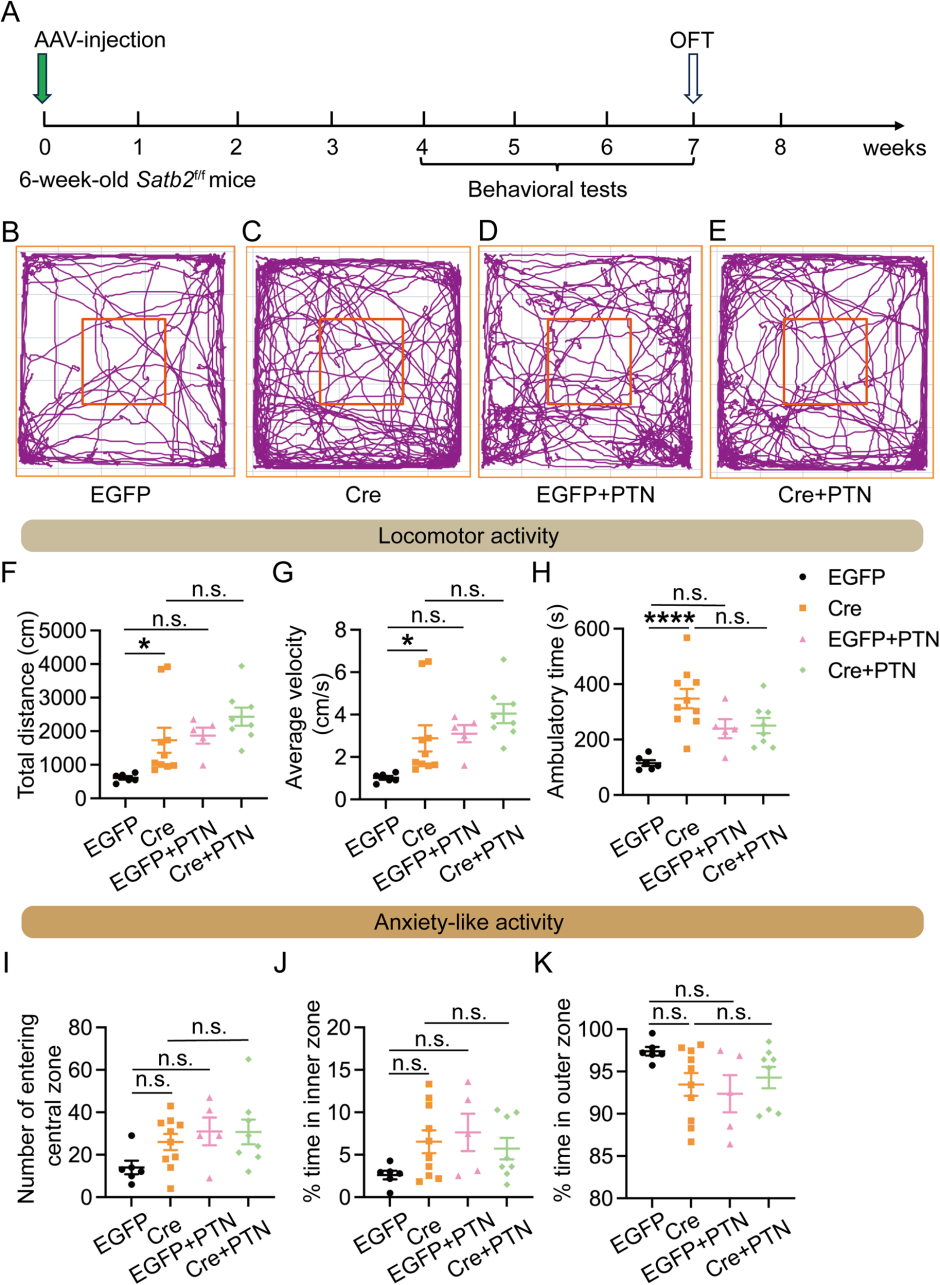
Deletion of *Satb2* in CA1 causes hyperactivity. **A** The schedule for AAV injection and the open-field test. **B**–**E** Schematic diagrams illustrating the movement trajectories of different groups of mice in peripheral or central regions. **F**–**H** Quantification of locomotor activity in the open field test includes total distance (**F**), average velocity (**G**), and ambulatory time (**H**) for the four groups of mice. **I**–**K** Quantification of anxiety-like behaviors in the open field test comprises the number of entries to the center zone (**I**), the proportion of time spent in the inner zone (**J**), and the proportion of time spent in the outer zone (**K**) for the four groups of mice. The sample sizes are as follows: *n* = 6 mice (four males, two females) for AAV-EGFP, *n* = 10 mice (six males, four females) for AAV-Cre, *n* = 5 mice (four males and one female) for AAV-EGFP + PTN, and *n* = 8 mice (six males and two females) for AAV-Cre + PTN. The data are presented as mean ± SEM. Statistical analysis was performed using one-way ANOVA with Dunnett’s multiple comparisons test. Statistical comparisons: EGFP vs Cre, *P < 0.05, ****P < 0.0001, n.s. non-significant; Cre vs Cre + PTN, n.s. non-significant; EGFP vs EGFP + PTN, n.s. non-significant, OFT open field test.

Previous research has identified a hyperactivity phenotype in *Satb2*^*Emx1*^ conditional knockout (CKO) mice [11], with *Emx1*-Cre activation beginning as early as embryonic day 10.5 [35]. To investigate the impact of Satb2 reduction on hyperactivity in adult mice, *Satb2*^flox/flox^ mice underwent an open-field test seven weeks post viral injection (Fig. 4A). The study revealed a significant increase in total distance traveled by AAV-Cre-infected mice compared to AAV-EGFP-infected mice (Fig. 4B, C, F). Moreover, AAV-Cre-infected mice exhibited higher average velocity and ambulatory time than AAV-EGFP-infected mice (Fig. 4G, H), indicating hyperactivity resulting from Satb2 loss in the CA1 region in adult mice.

Anxiety-like behaviors in a novel environment were also evaluated using the open-field test. The analysis demonstrated that the number of entries and time spent in the central zone or in the outer zone were comparable between AAV-Cre-infected and AAV-EGFP-infected mice (Fig. 4B, C, I, J, K). These findings suggest that *Satb2* deletion in CA1 has no effect on anxiety-like behaviors in adult mice.

### Loss of Satb2 in the hippocampal CA1 causes morphological changes of pyramidal neurons in adult mice

**Fig. 5:**
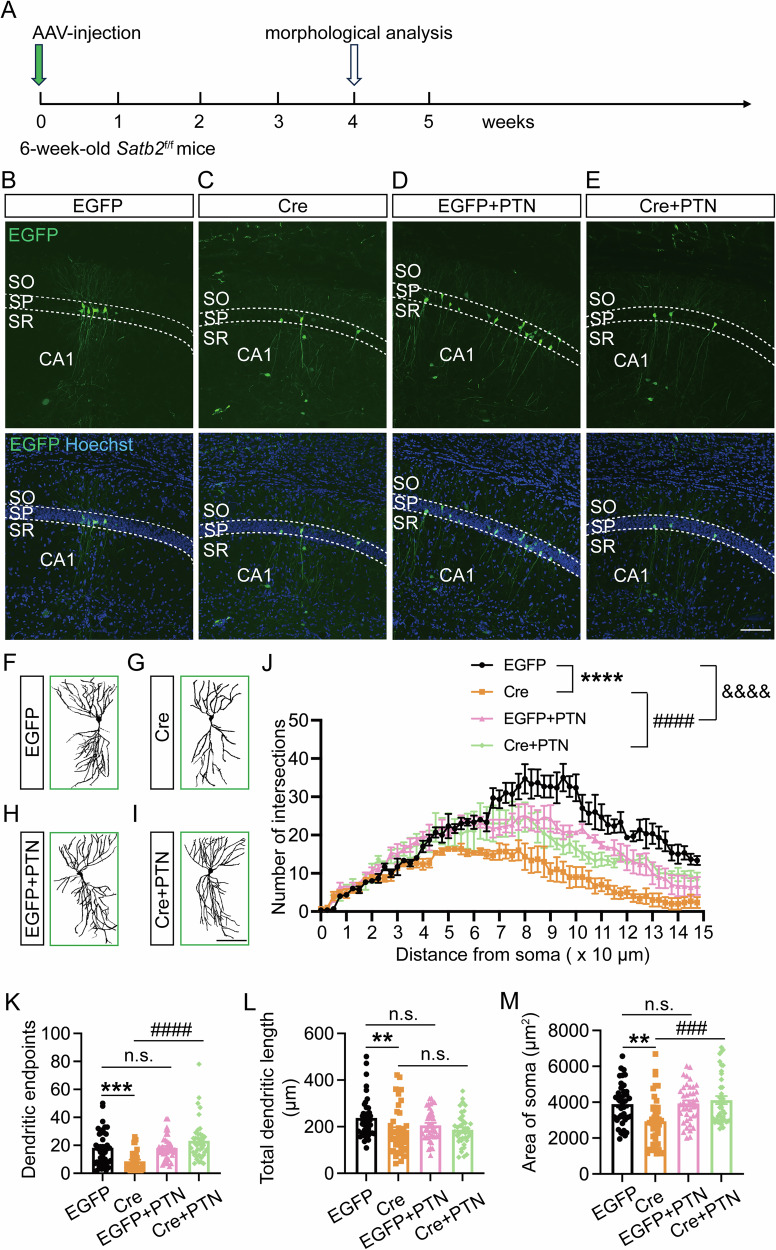
Deletion of *Satb2* in CA1 causes reduced soma size and simplified dendritic arborization in pyramidal neurons, which can be alleviated by overexpression of PTN. **A** Schematic illustrating the experimental design and timeline for morphological analysis. **B**–**E** Representative images showing sparsely infected pyramidal neurons in the hippocampal CA1 across four groups. Scale bar = 100 μm. **F**–**I** Representative traces depicting dendritic morphology of CA1 pyramidal neurons in the four groups. Scale bar = 100 μm. **J** Statistical data from Sholl analysis on dendritic branching of pyramidal neurons. **K** Quantification of dendritic endpoints of cells. **L** Measurement of total dendritic length. **M** Evaluation of soma size. *n* = 40 neurons from 3 mice per group. Data were presented as mean ± SEM. A two-way ANOVA with Sidak’s multiple comparisons test (**J**) or a one-way ANOVA with Dunnett’s multiple comparisons test (**K**–**M**) was used: EGFP vs Cre, **P < 0.01, ***P < 0.001, ****P < 0.0001; Cre vs Cre + PTN, ^###^*P* < 0.001, ^####^*P* < 0.0001; EGFP vs EGFP + PTN, ^& & & &^*P* < 0.0001; n.s. non-significant.

A close association exists between learning memory and neuronal dendritic morphology [36–38]. To examine the potential morphological effects of reducing Satb2 in the CA1 on pyramidal neurons, we conducted injections of diluted AAV-EGFP or AAV-Cre to infect the neurons, followed by morphological analysis after four weeks (Fig. 5A). This method allowed for sparse infection of pyramidal neurons by the diluted AAVs (Fig. 5B, C), enabling labeling with EGFP for easy tracing of neuronal morphology (Fig. 5F, G). Our findings revealed a significant reduction in soma size in AAV-Cre-infected neurons compared to AAV-EGFP-infected neurons (Fig. 5M). Additionally, AAV-Cre-infected neurons exhibited a notable decrease in the number of dendrite intersections, dendritic endpoints, and total dendritic length in comparison to AAV-EGFP-infected neurons (Fig. 5J–L). These results indicate that Satb2 depletion in CA1 pyramidal neurons leads to diminished cell somas and simplified dendritic branching in adult mice.

### Satb2 binds to genomic loci of PTN and positively regulates its expression in adult mice

**Fig. 6:**
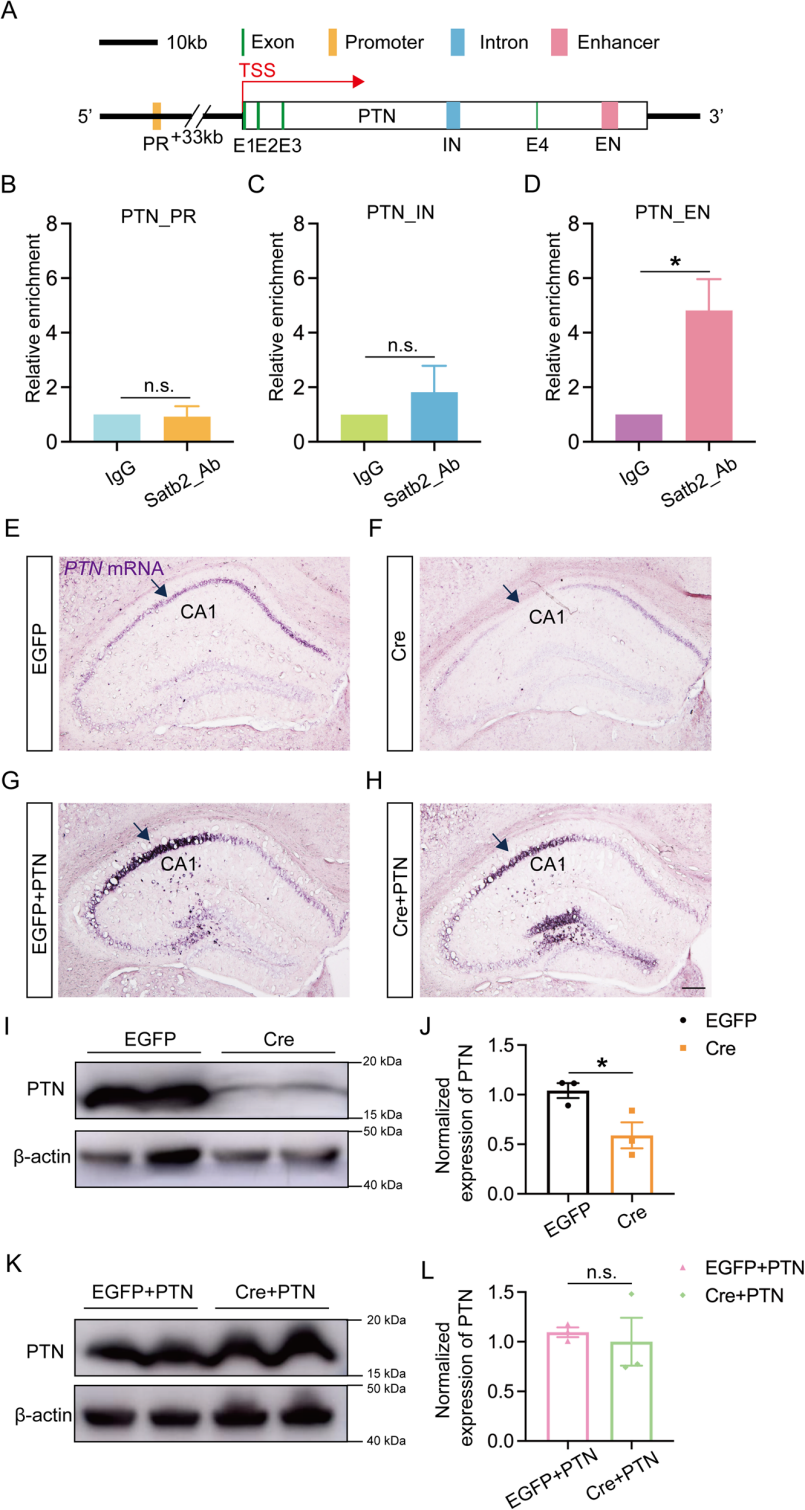
Satb2 binds to genomic loci of PTN and positively regulates its expression in the hippocampus of adult mice. **A** Diagram of potential Satb2-binding sites (PR, IN, and EN) in PTN genomic locus. (**B**–**D**) CUT&RUN analysis revealed a specific enrichment of Satb2 with PTN_EN, but not with PTN_ PR or PTN_ IN. Data were obtained from three independent experiments using hippocampal tissues (*n* = 10 for each experiment). **E**–**H** Representative images of *PTN* mRNA expression in the hippocampal CA1 region were shown for four groups via in situ hybridization. Scale bar = 100 μm. **I** Western blot analysis demonstrated a reduction in PTN levels in hippocampal tissue following *Satb2* deletion. **J** PTN protein levels were normalized to β-actin in both AAV-EGFP and AAV-Cre groups. **K** Western blots confirmed PTN overexpression in hippocampal tissue. **L** Protein levels of PTN are normalized to β-actin in AAV-EGFP + PTN and AAV-Cre + PTN groups. *n* = 3 mice for each group. Data were presented as mean ± SEM. Statistical analysis was performed using an unpaired two-tailed Student’s *t*-test: *P < 0.05; n.s. non-significant.

Previous research has shown that Satb2 acts as a transcription factor that binds to the regulatory regions of target genes to modulate their transcription [18, 19, 39, 40]. PTN, a secreted growth factor, is expressed in the hippocampus [41]. Recent studies have linked PTN to sleep deprivation-induced cognitive decline [42] and its potential in mitigating age-related cognitive impairment [17]. Given these findings, it is plausible that PTN may be a direct downstream target of Satb2. To investigate this hypothesis, a CUT&RUN-qPCR assay was conducted on hippocampal tissue samples. Analysis of previous Satb2 ChIP-seq data [43, 44] revealed three potential genomic binding sites of Satb2 within the PTN gene, namely in the promoter (PTN_PR), intron (PTN_IN), and enhancer (PTN_EN) regions (Fig. 6A). Results demonstrated that the enrichment levels at PTN_ PR and PTN_ IN were similar between Satb2 antibody (Satb2_Ab) and IgG controls (Fig. 6B, C), while there was a significantly higher enrichment of Satb2 at the PTN_EN locus compared to the IgG control (Fig. 6D). These findings suggest that Satb2 may regulate PTN expression by binding to the PTN_EN locus.

The role of Satb2 in regulating PTN expression in the hippocampus of adult mice was investigated by performing viral injections to knockout *Satb2* in the CA1 region, followed by in situ hybridization to assess PTN mRNA levels. The results revealed an enrichment of PTN mRNA in the CA1 of AAV-EGFP-infected mice, whereas its expression was significantly reduced in AAV-Cre-infected mice (Fig. 6E, F). Furthermore, Western blot analysis was conducted to evaluate PTN protein levels in hippocampal tissues from AAV-injected mice, showing a notable decrease in PTN expression in AAV-Cre-infected mice compared to AAV-EGFP-infected mice (Fig. 6I, J and Supplementary Fig. 1A). These findings collectively suggest that Satb2 potentially interacts with the genomic loci of PTN and that its deletion leads to a downregulation of PTN expression, indicating a positive regulatory role.

### Overexpression of PTN rescues morphological changes caused by the loss of Satb2 in the CA1

Having established PTN as a downstream factor of Satb2, we investigated the potential reversal of morphological changes and behavioral deficits in AAV-Cre-infected mice through PTN overexpression in the CA1 region. AAV-PTN was administered alongside AAV-EGFP or AAV-Cre in adult *Satb2*^flox/flox^ mice (Fig. 1B). PTN expression was evaluated using in situ hybridization and Western Blots, revealing elevated PTN mRNA levels in the CA1 of both AAV-EGFP + PTN and AAV-Cre+PTN-infected mice compared to AAV-EGFP controls (Fig. 6E, G, H). Western blot analysis confirmed similar PTN protein levels in AAV-EGFP + PTN and AAV-Cre+PTN-infected mice (Fig. 6K, L and Supplementary Fig. 1B).

Subsequently, neuronal morphology was examined post-PTN overexpression in the CA1. AAVs were injected into the CA1, and sparsely infected pyramidal neurons were traced for morphological assessment (Fig. 5D, E, H, I). Neurons with Satb2 loss and PTN overexpression exhibited larger soma size than those with Satb2 loss alone (Fig. 5M). Moreover, AAV-Cre+PTN-infected neurons showed increased dendrite intersections and endpoints compared to those with Satb2 loss alone (Fig. 5J, K), with comparable total dendritic length between the two groups (Fig. 5L). Neurons overexpressing PTN exhibited reduced dendritic complexity in comparison to those overexpressing EGFP alone (Fig. 5J). These findings suggest that PTN overexpression can partially rescue morphological changes in CA1 pyramidal neurons induced by Satb2 loss.

### Overexpression of PTN rescues cognitive dysfunction caused by the loss of Satb2 in the CA1

We investigated whether Satb2-dependent cognitive functions are dependent on PTN expression. Adult *Satb2*^flox/flox^ mice were injected with a combination of AAV-Cre+PTN or AAV-EGFP + PTN into the CA1. AAV-Cre+PTN-infected mice showed a significant preference for the novel object in the NOR test, unlike AAV-Cre-infected mice (Fig. 2C, D). AAV-Cre+PTN-infected mice exhibited a significantly increased memory index in the spontaneous alternation test compared to AAV-Cre-infected mice (Fig. 2F). In the spatial memory test, AAV-Cre+PTN-infected mice showed a significant increase in entries and time spent in the novel arm compared to AAV-Cre-infected mice (Fig. 2H, I). In the social preference test, the social interaction index was comparable between AAV-Cre+PTN-infected mice and AAV-Cre-infected mice (Fig. 3E). However, AAV-Cre+PTN-infected mice displayed a significant preference for S2 over S1 in the social recognition test, unlike AAV-Cre-infected mice (Fig. 3F), and the social interaction index was significantly higher in AAV-Cre+PTN-infected mice compared to AAV-Cre-infected mice (Fig. 3H). These findings indicate that PTN overexpression in Satb2-deleted neurons in the CA1 region ameliorates cognitive and social deficits in adult mice.

Locomotor activity and anxiety-like behaviors were assessed in AAV-EGFP + PTN-infected and AAV-Cre+PTN-infected mice (Fig. 4D, E). Results indicated no significant differences in total traveled distance, average velocity, and ambulatory time between AAV-Cre-infected and AAV-Cre+PTN-infected mice (Fig. 4F–H). Similarly, the number of entries to the central zone and time spent in the inner and outer zones were comparable between AAV-Cre-infected and AAV-Cre+PTN-infected mice (Fig. 4I–K). Additionally, locomotor activity and anxiety-like behaviors were also observed with no difference between AAV-EGFP + PTN-infected and AAV-EGFP-infected mice (Fig. 4F–K). These findings suggest that Satb2 modulates locomotor behaviors in a PTN-independent manner.

## Discussion

**Fig. 7:**
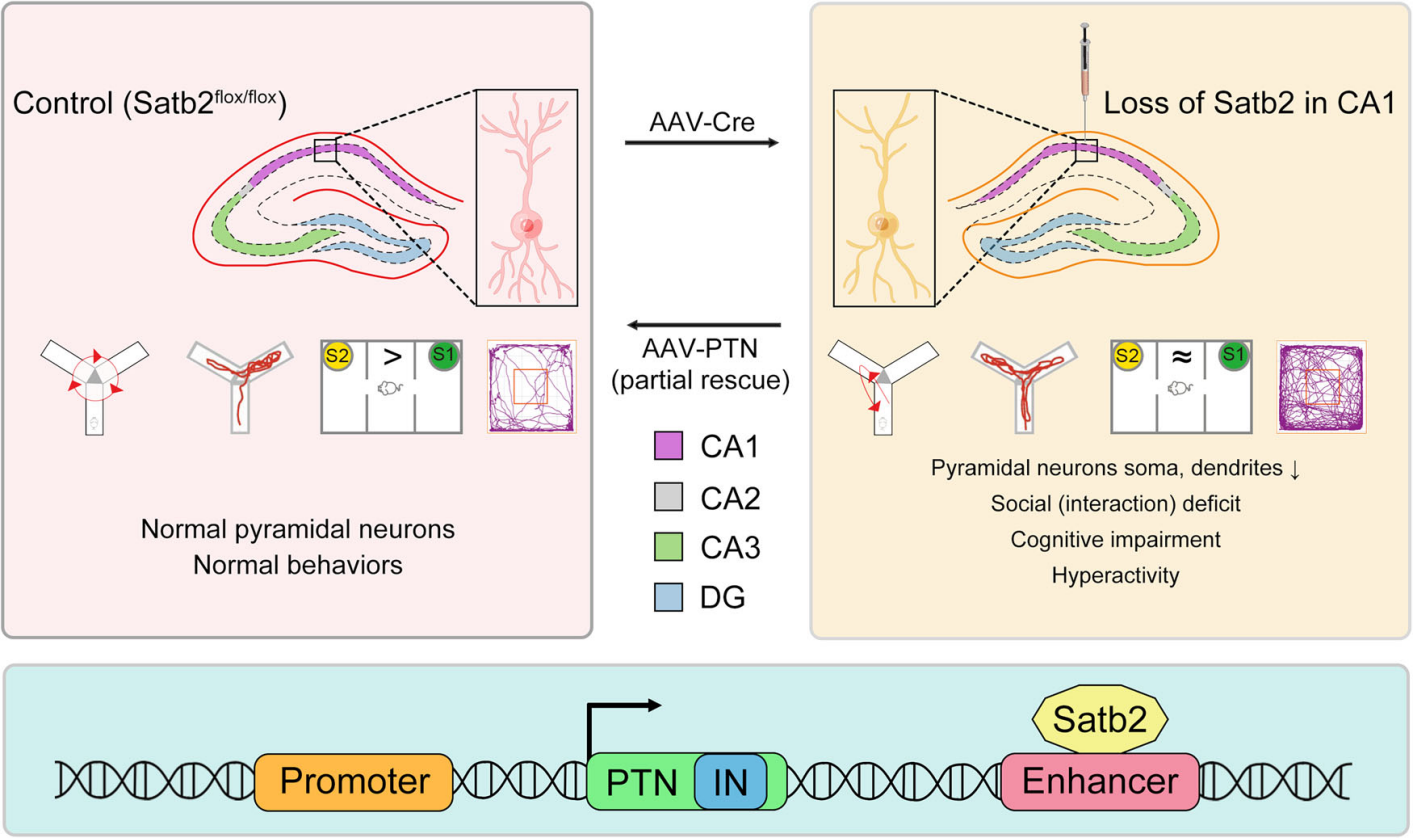
Schematic summary of this study. This figure summarizes the major findings of this study.

This study investigates the role of hippocampal Satb2 in adult mice, elucidating a novel mechanism underlying Satb2-dependent cognitive function in adults (Fig. 7). Deletion of *Satb2* in the CA1 region of adult mice significantly impairs cognitive function, leading to deficits in recognition memory, spatial working memory, reference memory, and social recognition behavior (Figs. 2, 3). Additionally, alterations in locomotor but not anxiety-like behaviors were also observed (Fig. 4). Furthermore, a reduction in soma size, dendritic length, and complexity of pyramidal neurons in the hippocampal CA1 was identified (Fig. 5). Lastly, it was demonstrated that Satb2-dependent cognitive function is contingent on the expression of PTN (Fig. 6).

Knockout of *Satb2* using the Emx1-Cre mouse line in a previous study resulted in impaired spatial learning and memory, abnormal social behavior, hyperactivity, and reduced anxiety-like behaviors [11]. This study utilized AAV-Cre injection into the CA1 region of the hippocampus in adult mice to bypass the developmental role of Satb2. The AAV-Cre-infected mice exhibited similar behavioral phenotypes, indicating the importance of Satb2 not only in development but also in cognitive function in the mature brain. Unlike previous studies targeting the entire cortex and hippocampus, this study focused on the unique role of Satb2 in the hippocampal CA1. While embryonic knockdown or knockout of *Satb2* in cortical neurons led to soma clumping and dendritic fasciculation [18, 31], these were not observed in CA1 pyramidal neurons in this study. In addition, cultured cortical neurons with *Satb2* knockdown showed increased dendritic complexity [31], contrasting with the reduced dendritic length and endpoints in CA1 pyramidal neurons lacking Satb2 in this study. This discrepancy may stem from differences in gene deletion timing or inherent distinctions between cortical and hippocampal neurons.

PTN, a secreted growth factor, acts as a crucial neuromodulator with diverse neuronal functions. Its expression is widespread in the central nervous system during early development [45], while in the adult brain, it is constitutively expressed in the cerebral cortex, hippocampus, cerebellum, olfactory bulb, and striatum [41, 46–48]. Despite the well-established role of PTN in cognitive function, the transcriptional regulation of this gene remains incompletely understood. Our findings in the hippocampal CA1 region reveal that Satb2 regulates PTN expression for cognitive function. Notably, overexpression of PTN fails to reverse hyperactivity behaviors resulting from Satb2 loss in the CA1 (Fig. 4F–H), indicating complex actions of PTN in behavior regulation. In the hippocampus, the receptor protein tyrosine phosphatase ζ (RPTPζ), a PTN receptor, is located at the postsynaptic membrane of adult pyramidal neurons [49], influencing spatial learning and long-term memory [50], as well as dendritogenesis and synaptogenesis of hippocampal neurons in vitro [51]. Thus, PTN signaling through RPTPζ may modulate hippocampal plasticity during learning by regulating dendritogenesis and synaptogenesis. Similarly, the anaplastic lymphoma kinase (ALK) receptor, another type of PTN receptor, is expressed in the adult mammalian hippocampus and is implicated in neurogenesis, memory, and learning [52], as well as basal hippocampal progenitor proliferation, with its deficiency leading to behavioral test alterations. Future investigations may focus on identifying the receptor for PTN in Satb2-dependent cognitive function.

In conclusion, we have shown that the transcription factor Satb2 plays a crucial role in shaping the morphology of pyramidal neurons in the hippocampal CA1 region, thereby influencing cognitive function. Further, our findings indicate that the Satb2-PTN axis may be a significant contributor to the pathogenesis of SAS, suggesting opportunities for the development of innovative therapeutic approaches in future investigations.

## Supplementary information

Supplementary figures and legends

Reproducibility checklist

## Data Availability

All data are available from the corresponding authors upon reasonable request.
