# Supplementary material for: Hippocampal Satb2 regulates the cognitive function of adult mice through pleiotrophin
Source: Cell Death Dis. 2026 May 6;17(1):601. doi: 10.1038/s41419-026-08820-z (PMC13316106; doi:10.1038/s41419-026-08820-z)
Supplement: Supplementary file 1 — Supplementary figures and legends [file 41419_2026_8820_MOESM1_ESM.docx]

**Supplementary figures and legends**


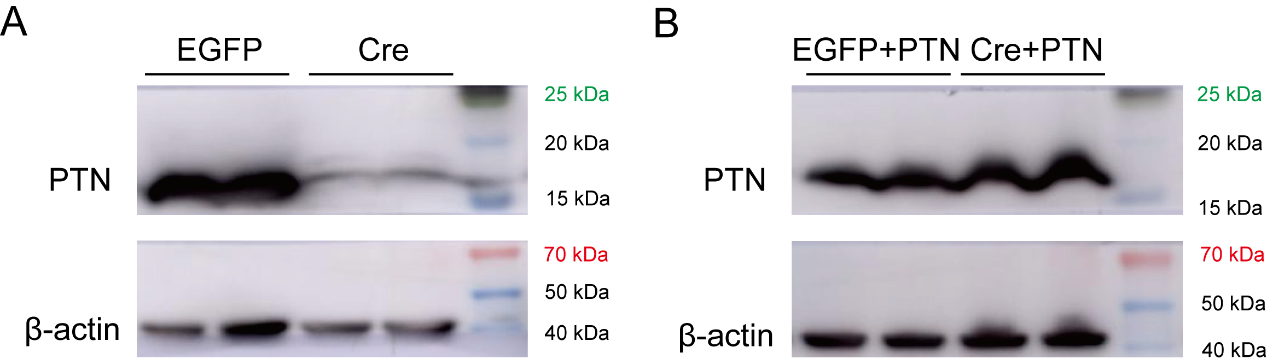


**Fig. S1** Full-length uncropped pictures of the Western blots presented in the figures.
